# Catalytic Production of Jet Fuels from Biomass

**DOI:** 10.3390/molecules25040802

**Published:** 2020-02-12

**Authors:** Manuel Antonio Díaz-Pérez, Juan Carlos Serrano-Ruiz

**Affiliations:** Materials and Sustainability Group, Department of Engineering, Universidad Loyola Andalucía, Avda. de las Universidades s/n, 41704 Dos Hermanas, Seville, Spain; madiaz@uloyola.es

**Keywords:** jet fuels, biomass, hydrocarbon fuels, heterogeneous catalysis, alcohols to fuels

## Abstract

Concerns about depleting fossil fuels and global warming effects are pushing our society to search for new renewable sources of energy with the potential to substitute coal, natural gas, and petroleum. In this sense, biomass, the only renewable source of carbon available on Earth, is the perfect replacement for petroleum in producing renewable fuels. The aviation sector is responsible for a significant fraction of greenhouse gas emissions, and two billion barrels of petroleum are being consumed annually to produce the jet fuels required to transport people and goods around the world. Governments are pushing directives to replace fossil fuel-derived jet fuels with those derived from biomass. The present mini review is aimed to summarize the main technologies available today for converting biomass into liquid hydrocarbon fuels with a molecular weight and structure suitable for being used as aviation fuels. Particular emphasis will be placed on those routes involving heterogeneous catalysts.

## 1. Introduction

Fossil fuels (petroleum, natural gas, and coal) supply most of the energy consumed worldwide. According to recent data from the U.S. Energy Information Administration [1], 80% of the energy consumed worldwide in 2018 was obtained from fossil fuels, and this share is expected to decrease only slightly (70%) by 2050. This dependence is even more drastic for the transportation sector, which relies almost entirely (95%) on petroleum. The transportation sector is a fast growing sector, particularly in developing countries. One third of the energy consumed worldwide is used to transport people and goods by burning liquid hydrocarbon fuels (gasoline, diesel, and jet fuels) produced from petroleum. The massive utilization of fossil fuels is, however, associated with important environmental (e.g., greenhouse gas emissions and associated climate change) and geo-political issues [2], and important directives have been issued to promote a progressive replacement of fossil fuels with more equally distributed and less contaminant renewable energies [3]. While coal and natural gas are to be replaced by solar and wind in the production of electricity, petroleum can only be substituted with biomass, which is the only source of renewable carbon available on Earth. Current strategies to decarbonize the transportation sector include the use of gas (hydrogen) and liquid biofuels (bioethanol and biodiesel) and also the implementation of electric vehicles (EVs) driven by Li-ion batteries. Despite the tremendous efforts carried out in the past years, the penetration of these alternative fuels and EVs in the transportation sector has been rather limited. Reasons for this low penetration include the lack of infrastructure for new fuels (particularly for hydrogen and EVs), compatibility issues with the current hydrocarbon-based infrastructure (e.g., blending wall of ethanol in gasoline), lower fuel mileage, and higher cost of the vehicles or fuels, among others. Additionally, the penetration of these new technologies in the transportation sector has not been homogeneous and depends highly on the type of vehicle. For instance, light duty vehicles are expected to be progressively replaced with bioethanol-driven or EVs in the near future, while heavy-duty vehicles can currently run on diluted B blends (i.e., mixtures of biodiesel and fossil diesel fuels) without the need for significant engine changes. Neither of these solutions are suitable for air vehicles. The stringent requirements of jet fuels (e.g., use at very low temperatures and high energy density, among others) makes biodiesel (a mixture of fatty acid methyl esters with high cloud points) unsuitable for these applications. In addition, current Li-ion batteries are far from having the required characteristics to propel heavy jets. Current batteries are very heavy and do not contain enough energy to get most planes off the ground. Although the energy density of batteries has been steadily increasing during the last decade, jet fuels still provide 43 times more energy than a battery with the same weight [4]. Within the next decades, the aviation sector will continue to rely almost completely on liquid hydrocarbon fuels, which already provide excellent energy density and cold-flow properties. Thus, the only viable alternative to reduce the dependence of this sector on petroleum lies in producing liquid hydrocarbon fuels chemically identical to those currently used from biomass, the so called green hydrocarbons [5]

The aviation sector consumes nearly 2,000 million petroleum barrels every year and is responsible for an important fraction of the greenhouse gas emissions released to the atmosphere. Thus, 669 million tons of CO_2_ are released annually to the atmosphere by the aviation sector [6], which accounts for an important fraction of the overall CO_2_ emissions worldwide. Air travel demand is expected to increase over the next 30 years at a rate of 3–5% for passengers and 10% for goods, which will result in jet fuel consumption doubling and CO_2_ emissions increasing six times by 2050. With the aim to reduce the dependence of the aviation sector on petroleum and mitigate the associated greenhouse gas emissions, governments are issuing directives to promote the use of jet fuels produced from biomass (i.e., biojet fuels). In this sense, the European Commission, through the “*European Advanced Biofuels Flightpath*”, has established ambitious objectives of production for the next several years [7], while the international aviation industry has committed to reducing its greenhouse gas emissions by 50% before 2050. In 2011, the U.S. Department of Agriculture and the U.S. Department of Energy agreed to invest more than 500 million dollars to produce drop-in aviation fuels for military and commercial applications [8]. To achieve these goals, it is imperative to develop technologies allowing for the transformation of biomass into liquid hydrocarbon fuels chemically identical to those currently used in the aviation sector. The present review is aimed to summarize the main technologies available today for this purpose. These technologies will be analyzed from the viewpoints of process complexity, economics, greenhouse gas emissions, and commercial status. Particular emphasis will be placed on those technologies involving catalytic processes.

## 2. Composition and Specifications of Jet Fuels

**Figure 1:**
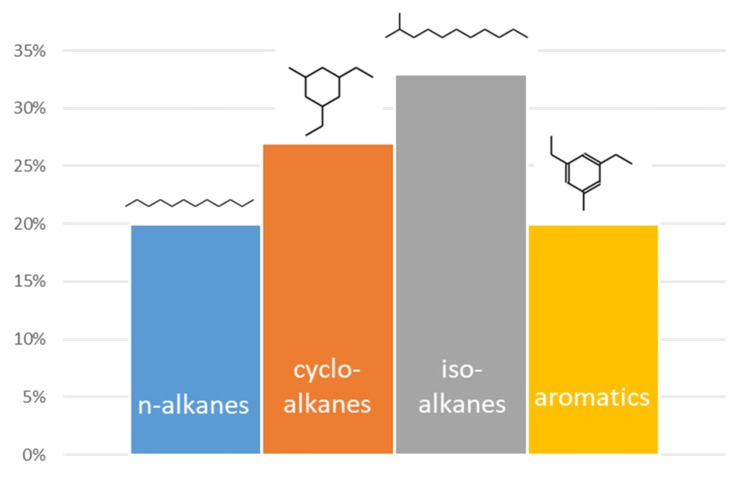
Chemical composition of jet fuel Jet A-1.

The most common jet fuel (Jet A-1) is a complex mixture of hydrocarbons in the C_9_–C_16_ range. These hydrocarbons belong to four families, namely linear alkanes; slightly branched alkanes or isoalkanes; cycloalkanes/naphtanes; and aromatics (Figure 1). Normally, 80% of the fuel is comprised of alkanes (linear, iso, and cyclic), while aromatics (substituted benzenes and naphthalenes) account for the remainder. The relative content of these four families of compounds varies depending on the season and location. Jet fuels have very strict requirements compared to road transportation fuels. Thus, the specifications of jet fuels cover physico-chemical characteristics such as freeze point, energy density, flash point, viscosity, flammability, combustion performance, sulfur content, density, and acidity, among others. These stringent specifications are detailed in ASTM D1655-09 [9]. The physical and chemical properties of any new jet fuel produced from biomass need to be carefully characterized before the fuels are certified for operation. Both the carbon chain length and the relative content of the four families of hydrocarbons detailed above are carefully controlled to provide the jet fuel with desired properties [10,11]. In this sense, jet fuels must have high flash points (to reduce fire hazard on board), low freeze points (to ensure good cold flow properties at high altitudes), high energy density (to minimize fuel storage room), and good sealing properties (to avoid fuel leakage on board), among others. For instance, linear alkanes are important to increase the energy density of the jet fuel while also improving its combustion performance, since they typically burn very cleanly [12]. Although iso-alkanes have high energy density, these hydrocarbons are mostly added to the jet fuel to improve its cold flow properties, since they have freezing points well below those of their linear counterparts. Cyclo-alkanes have a lower hydrogen to carbon ratio than linear and iso-alkanes and therefore provide less energy per mass unit. However, cyclo-alkanes are appreciated jet fuel components because they allow for increasing the density of the fuel while lowering its freezing point. Aromatics are important components of jet fuels. Despite negatively affecting the combustion performance of the fuel, a certain amount of aromatics (e.g., 20%) is mandatory since they promote swelling of elastomeric valves in fuel systems, thereby ensuring proper sealing [13,14]. Above this amount, aromatics do not provide beneficial effects to the jet fuel. Jet A-1 has a very low freezing point of −47 °C, which makes it suitable for winter or long international flights through polar routes.

## 3. Routes for the Production of Jet Fuels from Biomass

**Figure 2:**
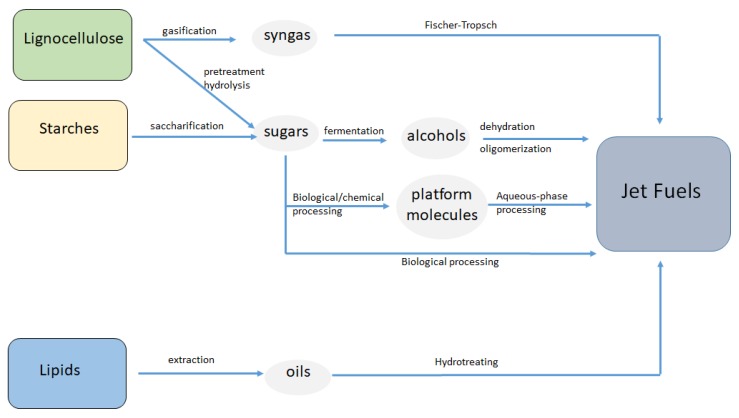
Routes for the conversion of biomass feedstocks (lignocellulose, starches, and lipids) into jet fuels. Species highlighted in grey correspond to less oxygenated intermediates, which are subsequently upgraded to hydrocarbons in the range of jet fuels by well-known catalytic and biological routes.

Despite both being natural carbon sources, petroleum and biomass have very different chemical compositions. While petroleum is composed of fully deoxygenated hydrocarbons with different molecular weights and levels of branching/cycling, biomass feedstocks (e.g., lignocellulose, starches, and lipids) are highly oxygenated. This different composition determines the processing strategies used to convert both resources into hydrocarbon fuels. Thus, the nature of petroleum feeds (hydrophobic, very volatile, and inert) obligates carrying out the conversion at high temperatures and in the gas phase. In contrast, biomass feeds are highly reactive and therefore require significantly lower temperatures than petroleum compounds. Petroleum derivatives are completely unfunctionalized and can therefore be used almost directly for the production of liquid transportation fuels (gasoline, diesel, and jet fuel) after simple fractional distillation and catalytic processing. These steps, required to adjust the molecular weight and structure of the hydrocarbons, do not involve profound chemical transformations. In contrast, the catalytic production of liquid hydrocarbon fuels from biomass is a complex process that involves deep chemical transformations through selective oxygen removal steps, followed by molecular-weight and structure adjustments. The oxygen removal steps (e.g., dehydration, hydrogenation, decarboxylation, C–O hydrogenolysis) are required to control the high reactivity of the biomass molecules, allowing the production of less reactive (yet active) intermediates. These intermediates are then more easily processed by well-known catalytic reactions such as Fischer–Tropsch, oligomerization dehydration, isomerization, and hydrogenation, which are aimed to increase the molecular weight of the intermediates and completely deoxygenate the final product. Figure 2 shows the main routes available today for converting biomass into jet fuels. In all cases, a first conditioning step is necessary to reduce the structural complexity of biomass and to produce oxygenated intermediates less complex and therefore more amenable for subsequent upgrading to jet fuels. These intermediates (oils, syngas, alcohols, and sugars/platform molecules) give name to the different routes for the conversion of biomass into jet fuels: oil to jet fuels (OTJ), gas to jet fuels (GTJ), alcohols to jet fuels (OTJ), and sugars/platform molecules to jet fuels (STJ), respectively. The most relevant aspects of these four routes will be described in the following subsections.

### 3.1. Oil to Jet Fuels

**Figure 3:**
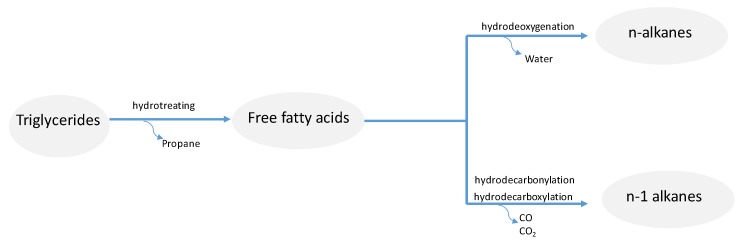
Scheme of the hydrotreating process for the conversion of TGs derived from vegetable oils into jet fuels.

Vegetable oils and related feedstocks can be transformed into liquid hydrocarbon fuels in the jet fuel range by a process denoted as catalytic hydrotreating [5]. This process allows the progressive removal of oxygen from the biomass feeds by performing catalytic processes at moderate to high temperatures (250–400 °C) and under high pressures of hydrogen (20–100 bar). The resulting fuel is denoted as hydroprocessed renewable jet (HRJ). Vegetable oils are rich in triglycerides (TGs), the natural components of fats and oils. TGs are formed by esterification of three fatty acids with glycerol. As shown in Figure 3, TGs first undergo saturation of the C=C double bonds present in the carbon chain followed by C–O hydrogenolysis to release the corresponding free fatty acids and a molecule of propane. The resultant free fatty acids can undergo progressive removal of oxygen by tandem hydrogenation/dehydration reactions (hydrodeoxygenation, HDO) to generate n-alkanes through the intermediate formation of the corresponding aldehydes and alcohols. In the case of HDO, oxygen is removed in the form of water. A fraction of the free fatty acids can also undergo decarbonylation/decarboxylation reactions (hydrodecarbonlylation/decarboxylation, HDC) leading n-alkanes with n-1 carbon atoms, with oxygen being removed as CO or CO_2_, respectively. Thus, HDC generates HRJ fuels at slightly lower carbon yields compared to HDO, although HDC allows for a reduction in the hydrogen consumption compared to the HDO route [15]. As indicated in the previous section, n-alkanes are valuable jet fuel components with high energy density and excellent combustion characteristics, although they have poor cold flow properties. To overcome this issue, the hydrotreating process typically contains an isomerization unit to generate iso-alkanes or slightly branched alkanes (Figure 3), having significantly lower pour and cloud points than their linear counterparts. The carbon chain length of the jet fuel is determined by the composition of the feedstock. The most relevant vegetable oils (e.g., canola, soybean, rapeseed, palm, corn, camelina, jatropha) are composed of C_16_ or C_18_ fatty acids so that hydrocarbons resulting from the hydrotreating process are typically within the C_15_–C_18_ range. Thus, apart from isomerization, a certain degree of hydrocracking is necessary to adjust the carbon length to the jet fuel range (C_9_–C_16_). Isomerization and hydrocracking processes are normally carried out in the same reactor over acidic catalysts. 

Catalysts used for the hydrotreating of vegetable oils include noble metal-supported materials and, to a greater extent, alumina-supported metal (e.g., Co, Mo, Ni, W) sulfides similar to those used in the petrochemical industry for the removal of heteroatoms such as nitrogen or sulfur [16,17,18,19]. The acid sites required to carry out the dehydration and HDC reactions are normally provided by the support [20], while hydrogenation reactions are carried out over the metal moiety. The strength of the acid sites and the porosity of the support need to be carefully controlled to avoid over-isomerization or uncontrolled cracking reactions leading to gaseous C_1_–C_4_ species and lower liquid alkanes out of the jet fuel range. Supports with narrow porosity (i.e., micropores) are not suitable for the hydrotreating of vegetable oils in light of the large size of the TGs. Thus, carriers with porosity in the mesopore range are preferred to avoid diffusion limitations and to minimize pore blocking by heavier hydrocarbons [21,22].

A large variety of feedstocks can be transformed into jet fuels by this route including fatty acids, TGs, vegetable oils [23,24,25], waste cooking oils [26,27], animal fats [28], and trapped greases [29], among others. Vegetable oils rich in fatty acids containing a high number of unsaturations such as camelina, jatropha, and algae-derived are particularly suitable for the production of jet fuels since these unsaturations facilitate the formation of branched hydrocarbons (with good cold-flow properties) during hydrotreating [30]. Camelina is a non-edible short-season crop with high oil content that has shown great potential to generate hydrocarbon mixtures meeting the stringent requirements for jet fuels and with low greenhouse gas emissions [31]. Camelina oil is currently produced in large amounts in the U.S. at low cost (e.g., 0.4–0.7 $/gal) [32]. Jatropha is one of the oil-producing crops with the highest oil yield. The high seed oil content (as high as 55%, dry weight basis) along with the possibility of using seed shells and husks for the production of value co-products increases the attractiveness of this plant for jet fuel production [33]. Jatropha can also grow in marginal lands, is drought resistant, and can maintain high-yield production for decades. Algae have a number of characteristics (rapid growth, high yield production, high oil content, possibility of co-producing valuable co-products) that have attracted the interest of many researchers worldwide for the production of biofuels [34]. Algae oils have been used as feedstocks for the production of jet fuels with very high yields (77%) and suitable iso-alkane content [35,36].

The hydrotreating process is a well-known catalytic technology, which has been exploited for several decades by the petrochemical industry to remove heteroatoms such as N and S from hydrocarbon feedstocks. This route has been successfully adapted to transform oxygenated feedstocks such as vegetable oils into hydrocarbon fuels in the diesel and jet range, and a large number of works have been described in the literature in the last few years [37,38,39]. While excellent results have been obtained with relatively pure vegetable oils and free fatty acids, hydrotreating of cheaper waste feedstocks is problematic due to the presence of impurities (e.g., S, N, alkali, P) that can deactivate the catalysts. Although hydrotreating allows the production of linear and slightly branched alkanes, this route normally fails to produce important components of jet fuels, namely cyclo-alkanes and aromatics (see Figure 1). As will be described below, this limitation is important in that it avoids the use of 100% HRJ in current aircraft. In this sense, important advances have been carried out in the last years. Jatropha oil was converted into HRJ over NiMo and NiW catalysts supported on a silicoaluminophosphate (SAPO) by Verma et al. [40]. A mixture of diesel and jet fuel alkanes were obtained in a flow reactor operating at high temperatures and pressures. Interestingly, a significant amount of aromatics (8%) were produced over the acidic SAPO support, which gives a competitive advantage to this process considering that HRJ are normally free of these compounds. This particular ability of SAPOs was also exploited by Rabaev et al. [41] to produce jet fuels (48% yield) with a significant content of aromatics (12%) from soybean oil. With the aim to improve the economics of the process, several researchers have attempted to carry out the conversion of vegetable oils into jet fuels in the absence of hydrogen over zeolitic materials [42,43]. Higher temperatures were required to achieve deoxygenation of the vegetable oils (e.g., 550 °C), which resulted in lower alkane yields and faster deactivations. These results suggest that a minimum amount of hydrogen seems to be required to prevent the formation of easily polymerized unsaturated intermediates. The utilization of water under near supercritical conditions as a reaction medium allowed Fu and co-workers to carry out the HDC of fatty acids with high selectivity and without the need of external hydrogen [44]. However, the utilization of high temperatures and pressures, along with the corrosive nature of water under these conditions, can be potentially problematic and increase the cost of the fuel.

The economics of the OTJ route has been analyzed in the literature by Pearlson et al. [45]. Minimum selling prices ranging from 4.4 to 5.1 $/gal were obtained for plant sizes ranging from 100 to 30 MM gal/yr, respectively. The cost of the feedstock and the hydrogen required to carry out the catalytic process accounted for most of the production costs. HRJ has been estimated to have higher capital costs than biodiesel [46]. However, these higher capital costs can be offset by commercializing co-products such as propane (resulting from the breaking of the triglyceride chain) or liquid petroleum gas generated by the cracking of higher hydrocarbons. The GHG emissions generated during the hydrotreating of vegetable oils to jet fuels have been quantified [47]. In general, HRJ achieves the important reduction of GHG emissions (30–70%) as compared to conventional jet fuels derived from petroleum. The extent of reduction is highly dependent on the land use, with crops growing in marginal lands providing high reductions. Hydrogen accounts for an important fraction of the GHG emissions of HRJ since this gas is typically obtained from natural gas. Reforming of the propane co-produced during the hydrotreating process can supply a large fraction of the hydrogen required for the process [48], thus also allowing an important reduction in CO_2_ emissions. 

The HRJ was the first biojet fuel conversion process approved for use in testing flights, and a good number of companies have developed commercial processes. The main HRJ producers are Neste Oil and Honeywell UOP, which have developed two-step (hydrotreating and hydroisomerization) trademark processes denoted as NExBTL and Eni-Ecofining, respectively. The NexBTL process allows the conversion of a range of vegetable oils and waste animal fats into jet fuels, meeting the ASTM standards while significantly reducing the carbon footprint and the NO_x_ emissions compared to conventional jet fuels [49]. Important companies such as Lufthansa are currently using jet fuels from Neste Oil in commercial flights [50]. The process from UOP allows the conversion of vegetable oils such as camelina, jatropha, and algae into jet fuels with excellent energy density and cold-flow properties. Since 2011, HJF can be used blended with regular jet fuel in amounts up to 50% *(v/v)* in commercial flights and military jets [51].

### 3.2. Gas to Jet Fuels

As shown in Figure 2, the GTJ route is comprised of two different processes, namely, biomass gasification to syngas (H_2_/CO), followed by Fischer–Tropsch conversion of this syngas into jet fuel hydrocarbons [52]. Gasification is a well-developed technology that allows conversion of virtually any carbon source (natural gas, coal, biomass) into a mixture of gaseous species (e.g., CO, H_2_, CO_2_, CH_4_) by applying a treatment at high temperatures under a carefully controlled oxidizing atmosphere (e.g., air, steam, oxygen). Control over the composition of the gas stream is difficult, with a large number of factors (biomass source, particle size, gasification conditions, and gasifier design) having an influence on the gasification performance [53]. Pure oxygen atmospheres, high temperatures (1300 °C), and high pressures lead to gas streams enriched in syngas [54]. The utilization of biomass feedstocks versus fossil carbon sources has two important implications for the downstream Fischer–Tropsch process. First, compared to coal or natural gas, biomass contains a larger number of impurities (N, Cl, S, tars, ashes, alkali) that are typically found accompanying CO and H_2_ in the gas stream. These impurities are particularly problematic because they deactivate Fischer–Tropsch catalysts. Second, the high oxygen content of biomass compared to fossil fuels results in syngas streams with H_2_/CO ratios (ca. 0.5) well below those required for the Fischer–Tropsch synthesis of hydrocarbons (ca. 2) [55]. Thus, before reaching the Fischer–Tropsch unit, the syngas delivered by the gasifier must be deeply cleaned and compositionally adjusted by performing additional cleaning and water gas shift (WGS) steps, respectively. These extra cleaning and WGS units significantly increase the complexity and cost of the GTJ process. Once the syngas is cleaned and compositionally adjusted, it can be fed to the Fischer–Tropsch reactor. The Fischer–Tropsch synthesis is a well-known highly exothermic industrial process that allows the conversion of syngas into a mixture of hydrocarbons with low carbon-chain selectivity (C_1_–C_50_) over metal-based catalysts at moderate temperatures and pressures [56]. The hydrocarbon distribution depends highly on both the operating conditions (temperature and pressure) and the catalyst composition. For instance, cobalt-based catalysts are particularly active in producing linear alkanes [57,58], while iron-based catalysts are more selective toward olefins and can operate at lower H_2_/CO ratios because of their significant WGS activity. Oxygenated compounds such as alcohols, aldehydes, and carboxylic acids are typically produced in the process along with the hydrocarbons. Addition of promoters such as K to Fe catalysts allows for an increase in the selectivity to jet fuel range hydrocarbons, as recently demonstrated by Martinez del Monte et al. [59]. The large amount of heat released during the Fischer–Tropsch process must be removed rapidly to avoid high temperatures in the reactor, which favor the formation of CH_4_ and lead to catalyst deactivation. In order to produce hydrocarbons in the jet fuel range (C_9_–C_16_), heavy hydrocarbons (waxes) can be produced first by operating at low temperatures (230 °C), followed by controlled cracking and isomerization steps to jet fuel components [60]. Thus, conventional petrochemical units such as hydrocracking, isomerization, and fractionation are normally required after the Fischer–Tropsch reactor to adjust the molecular weight and structure of the hydrocarbons to the jet fuel range. As in the case of the OTJ process, the jet-fuel obtained by this route lacks aromatics, thereby avoiding utilization of 100% renewable fuel and requiring blend mixtures. 

The high complexity of the GTJ route is an important factor limiting the commercialization of this technology. In fact, this process is only cost effective at large scales, which is counterproductive when using low energy density feeds such as biomass [38]. The chemical composition of biomass (i.e., high oxygen content and presence of impurities) increases the complexity of the GTJ route compared to routes starting from carbon or natural gas. Thus, according to Hileman et al. [61], the production costs of jet fuels obtained by biomass gasification are ca. 20% higher than those obtained by gasification of coal. The cost of the feedstock and the temperature of the gasifier accounts for a significant fraction of the production costs of the fuel. In this sense, higher temperature gasifiers are preferred since they allow the complexity of the syngas cleaning process to be reduced by providing cleaner gas streams and faster gasification kinetics. For a GTJ plant of 2000 tons per day, Anex et al. [62] reported significantly higher capital expenses when low-temperature gasifiers were used (610 vs. 500 M$ for gasifiers operating at 870 vs. 1300 °C, respectively). The GTJ route has higher capital costs than other thermochemical processes. The biomass pretreatment (mostly drying and mechanical particle size reduction), gasification, and syngas cleaning/conditioning units account for most of these capital expenses. In a recent study, Sahir et al. [63] analyzed the cost of producing liquid fuels from biomass/natural gas mixtures. Minimum selling prices ranging from 2.47 to 3.47 $/gasoline gallon equivalent (gge) were obtained in this study for a 50 million GGE/year facility. The addition of a hydrocracker allowed a substantial increase in the yield of diesel and jet fuel hydrocarbons, although this increase did not offset the higher capital expenses of the new unit. 

The CO_2_ generated by fossil fuel combustion in the gasifier and the emissions resulting from the Fischer–Tropsch reactor account for most of the GHG emissions of the GTJ process. Despite these emissions, the GTJ process achieved significant reduction of the GHG emissions (ca. 90%) compared to conventional jet fuel processes [64], provided that biomass can supply nearly half of the energy required to drive gasification and Fischer–Tropsch. GHG emissions as low as 2–10 g CO_2_/MJ have been reported for GTJ processes with corn stover, forest residues, and switchgrass as biomass feedstocks [65]. In this sense, the GTJ route is more attractive than the OTJ route, which has higher GHG emissions associated with the utilization of fertilizers to grow the oil plant. 

The high capital costs of the GTJ route have prevented large companies from investing in large facilities, and only test/pilot plants have been developed. Technical challenges associated with the handling of biomass and cleaning of the syngas have delayed (or even cancelled) industrial efforts to bring this technology to commercial status [66]. Despite these difficulties, a number of companies such as Red Rock Biofuels, Sasol, Fulcrum, and Total have built facilities with capacities of 30–48,000 tons/year of biomass (wood, municipal wastes), and some of them are intended to be operative this year [67,68,69,70]. Some of these companies have signed agreements with airline companies to supply renewable jet fuel. The GTJ fuel has been ASTM certified for commercial use blended with petroleum-derived jet fuel up to 50% [71] The lack of aromatics in the jet fuel is the main limitation to increasing the concentration of renewable jet fuel in these blends. Slight variations of the GTJ technology have been developed to overcome this limitation. These variations involve additional steps such as aromatization of a fraction of the syncrude or the addition of naphtha fractions enriched in monoaromatics. This new technology has been denoted as Fischer–Tropsch synthetic kerosene with aromatics (FT-SKA). This aromatic-containing jet fuel has been approved to be blended with Jet A1 conventional fuel in amounts up to 50 vol% [72]. 

### 3.3. Alcohol to Jet Fuels

**Figure 4:**
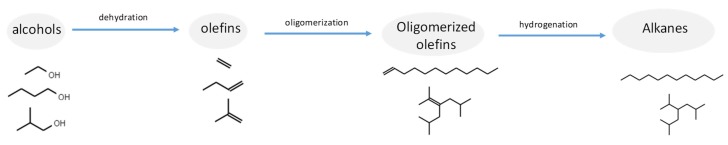
Scheme of the alcohols to jet process.

As shown in Figure 4, the ATJ route involves three main steps: (i) dehydration of the bioalcohol to the corresponding olefin; (ii) oligomerization of the olefins to a new oligomerized olefin, and (iii) hydrogenation of the oligomerized olefin to the saturated hydrocarbon product. As in the case of the GTJ route, these three individual steps are well-known and have been widely employed in the petrochemical industry, and the main barrier toward developing this technology lies in the integration. Alcohols typically used in the ATJ route include small C_2_ and C_4_ compounds such as ethanol (the most widely produced bioalcohol worldwide) and butanol (n-butanol and iso-butanol). These alcohols can be produced from biomass sugars by mature and simple microbial fermentation technologies similar to that used in beer and wine-making [73,74]. The extraction of sugars from the carbohydrate polymers is relatively easy with edible biomass feedstocks such as sugar cane or corn, in which case a simple treatment in hot water is enough to release the monomers. In the case of non-edible biomass (e.g., lignocellulose), sugar extraction is more problematic, and additional (and sometimes costly) pretreatments are required to break or weaken the lignin structure that surrounds the cellulose and hemicellulose polymers. Alcohols are produced in the biofermenters at low concentrations and near room temperature to allow microorganisms to survive.

Most of the bioethanol produced today is blended with gasoline at low concentrations (up to 15 vol%) to avoid engine compatibility and water-absorption issues [2]. This limitation in ethanol blending (i.e., blend wall) is currently causing important issues to absorb the growing bioethanol production. Ethanol has a number of drawbacks (e.g., high volatility, high water absorption, low flash point, corrosiveness, low energy density) that discourage its direct use as an aviation fuel [75]. The ATJ route represents an interesting approach to overcome these limitations by converting an oxygenated fuel into a mixture of hydrocarbons 100% compatible with the current transportation infrastructure. An excellent review on the catalytic routes available to convert ethanol into diesel and jet fuels was recently published by Eagan et al. [76]. Butanol is less miscible with water and, although it possesses higher energy density than ethanol, its energy content is still well below that of conventional jet fuel (33 vs. 42 MJ/kg). This latter issue is particularly relevant since it considerably reduces the flight range. 

As remarked above, alcohol dehydration and olefin oligomerization processes have been demonstrated at the commercial scale in the petrochemical industry. Ethanol can be readily dehydrated to ethylene over acidic catalysts such as silica-alumina, silicoaluminophosphates, zeolites, and heteropolyacids [77,78,79]. Complete conversion and nearly 100% selectivity toward ethylene were achieved over these materials at moderate temperatures (250 °C) and space velocities (2 h^−1^). Interestingly, dehydration of ethanol can be achieved even under aqueous environments over water-resistant carbon acidic catalysts at moderate temperatures [80], which opens the possibility of carrying out the dehydration of diluted aqueous ethanol solutions directly obtained from fermenters and without the need of expensive and energy intensive water removal steps. Dehydration of C_4_ alcohols also takes place smoothly over acidic catalysts, although obtaining a single olefin remains challenging. Isobutanol was readily converted into isobutylene over alumina catalysts at 325 °C and under water environments [81]. Apart from isobutylene, other C_4_ linear olefins such as n-butene and 2-butene were produced in minor amounts. The isobutylene/linear olefins was found to be mostly controlled by the catalyst composition and porosity [82]. N-butanol was dehydrated into 1-butene at silica-alumina with 95% selectivity at 380 °C and low space velocities [83].

The olefins resulting from the above dehydration processes can be oligomerized to higher olefins by well-known industrial processes using both homogeneous and heterogeneous catalysts. For instance, ethylene can be oligomerized to linear α-olefins using the commercial Ziegler–Natta catalysts [84]. This process has been industrially exploited by Chevron (one-step Ziegler–Natta) and INEOS (two-step Ziegler–Natta) to produce hundreds of thousands of ethylene oligomers annually. A third process has been developed by Shell using a different homogenous catalyst based on Ni–P [75]. Oligomerization of olefins can also be carried out over heterogeneous acidic catalysts such as sulfonic resins, solid phosphoric acid, or zeolites at moderate temperatures and pressures [85,86,87,88] Isobutene was oligomerized over Amberlyst to produce a distribution of C_8_–C_16_ olefins centered at C_12_ (70%) [89]. 1-hexene, produced by controlled trimerization of ethanol-derived ethylene, was oligomerized selectively over a metallocene catalyst [90]. The resultant C_12_ dimer showed an outstanding freezing point of −77 °C and a viscosity as low as 3.5 mPa s at −20 °C. 

Economic assessments of the overall ATJ process are lacking in the literature. While the cost of producing ethanol is well known and has been widely studied, the cost of the subsequent upgrading processes (dehydration, oligomerization, and hydrogenation) have not been evaluated yet. The same is valid for the GHG emissions, which are well known for alcohol production, but a complete evaluation of the overall process remains to be done. A good number of ATJ projects have been developed over the last years by partnering bioalcohol producers and companies able to perform the catalytic upgrading [38]. Most of these processes are at an early stage of development (laboratory and pilot). Gevo, an isobutanol producer, has partnered with BioChemtex to convert the bioalcohol into jet fuels in the C_12_–C_16_ range. Interestingly, a fraction of isobutanol can be used to produce valuable jet fuel components such as aromatics. This provides this process with a competitive advantage over other routes (e.g., OTJ and GTJ), producing exclusively aliphatic hydrocarbons [91,92]. Gevo is increasing the size of one of its plants to produce up to 10,000,000 gallons per year of ATJ, and it has partnered with Total to distribute renewable jet fuel in France and the rest of Europe [93]. Byogy Renewables has developed a process to transform ethanol into jet fuel, among other advanced biofuels. By means of a catalytic process, ethanol is converted into a mixture of long chain hydrocarbons, which is subsequently fractionated into jet fuel and gasoline fractions [94]. The process can be expanded to higher alcohols such as propanol and butanol and, as in the case of the Gevo process, aromatics can be produced as part of the catalytic process, opening the possibility of using 100% renewable fuel. In 2016, ASTM certified the ATJ fuel produced by Gevo from isobutanol in blends with conventional jet fuels up to 30 vol%. More recently, ATJ fuel produced from ethanol has approved Byogy fuel for use on commercial flights and increase the blend ratio limit to 50% for these fuels [95]. The potential integration of an ATJ process within a pulp mill facility has been recently analyzed and important synergies were identified including feed handling and power recovery systems [96]. The co-production of liquid fuels allowed the energy consumption of the pulp production plant to be reduced, although further economic analyses are required to determine the profitability of this integration.

### 3.4. Sugars and Platform Molecules to Jet Fuels

Sugars and sugar-derivatives other than alcohols can also serve as feedstocks for the production of jet fuels. The catalytic transformation of sugars and derivatives into liquid hydrocarbon fuels is a complex process that requires profound chemical changes. Thus, the sugars and platform molecules derived are highly oxygenated and contain a variety of functionalities (e.g., –OH, –C=O, and –COOH groups). Additionally, since these molecules are derived from sugar monomers, their maximum number of carbon atoms is limited to six. In contrast, aviation fuels are completely unfunctionalized and larger (C_9_–C_16_). Thus, in order to covert these molecules into jet fuels, oxygen removal steps (e.g., dehydration, hydrogenation, and hydrogenolysis) should be combined with C–C coupling reactions (e.g., aldol condensation, ketonization, and oligomerization). These chemical reactions are typically carried out in the aqueous phase since these molecules are highly soluble in water [97]. Therefore, one of the main challenges of this route lies in reducing the complexity of the process by decreasing the number of reactors required to carried out the transformations. Furthermore, the utilization of fossil hydrogen should be minimized during oxygen removal in order to reduce GHG emissions. Sugars can also be transformed into liquid hydrocarbon fuels (including jet fuels) by biological conversion with genetically modified microorganisms [5]. This route, however, is beyond the scope of the present paper.

**Figure 5:**
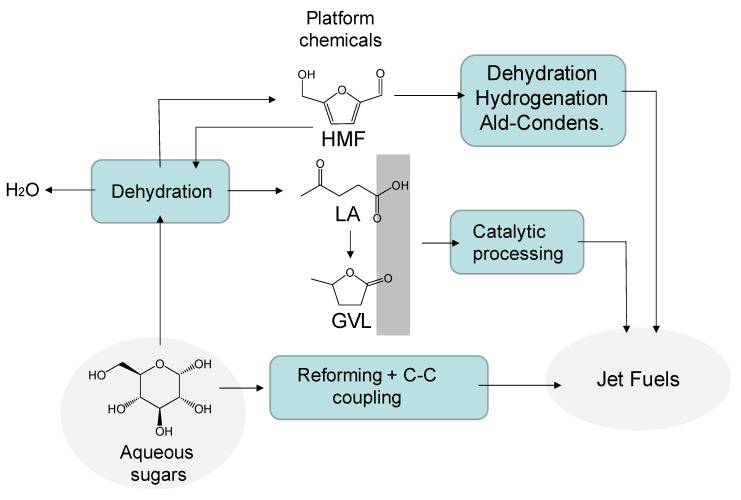
Scheme of the main routes for the conversion of sugars and platform molecules derived from them into jet fuels.

The main catalytic routes allowing for the conversion of sugars and derivatives into jet fuels are schematically shown in Figure 5.

Sugars can be converted into liquid hydrocarbon fuels with a structure and molecular weight suitable for jet fuel applications by a two-step cascade catalytic process developed by Kunkes et al. [98]. In a first step, sugars are partially deoxygenated (up to 80%) over a Pt–Re/C catalyst at moderate temperatures (e.g., 200 °C) to yield a mixture of monofunctional hydrocarbons in the C_4_–C_6_ range including acids, alcohols, ketones, and heterocycles. These monofunctionals are obtained in an organic phase that spontaneously separates from water, which facilitates separation from the aqueous phase. The Pt–Re/C catalyst allows oxygen removal by catalyzing C–C cleavage and C–O cleavage reactions. A fraction of the sugar is reformed over the bimetallic catalysts to produce hydrogen, which is supplied to the process. In a second step, each family of monofunctional compounds can be upgraded by C–C coupling reactions to the corresponding alkane product. For instance, ketones can be upgraded to (C_8_–C_12_) jet fuel components with low extents of branching by performing aldol-condensation over basic catalysts. Sugar-ketal derivatives such as 1,2:3,5-di-*O*-isopropylidene-α-d-xylofuranose were recently converted into cyclo- and iso-alkanes with molecular weights suitable for jet fuel applications by means of a single step catalytic process [99].

Furanics such as furfural (2-furaldehyde) and hydroxymethyl furfural (HMF) are readily obtained by the dehydration of aqueous sugars in the presence of mineral acids [100,101]. The presence of a carbonyl group allows these compounds to be used as platform molecules for the production of jet fuels. The process involves cascade dehydration, hydrogenation, and aldol-condensation reactions [102]. These furanics compounds can be condensed with other carbonyl compounds such as acetone or even with other furanic compounds to generate larger compounds with chain length appropriate for jet fuel applications [103]. Hydrogenation of the condensed abduct is required before obtaining the liquid hydrocarbon fuel with targeted molecular weights (C_9_–C_15_ for HMF and C_8_–C_13_ for furfural) [104]. Corma et al. [105] analyzed the potential of some furanic biomass derivatives to produce liquid hydrocarbon fuels. The molecular weight of these compounds was adjusted to the jet fuel range by aldol condensation and hydroxyalkylation reactions. Furanics can also serve as a source of aromatics for the jet fuel pool. As demonstrated by Chang et al. [106], dymethylfuran can be converted into p-xylene by cycloaddition with ethylene. This technology achieves the aromatic derivative with excellent yields (e.g., 90%). 

Levulinic acid (LA) is an important biomass-derived acid obtained by acid hydrolysis of cellulosic wastes [107]. Aqueous solutions of LA can be converted into jet fuels by a catalytic approach involving dehydration/hydrogenation and ketonization reactions [108,109]. In a first step, aqueous LA is hydrogenated to γ-valerolactone (GVL), a key intermediate in the production of jet fuels. Aqueous GVL is subsequently transformed into 5-nonanone with the intermediate formation of pentanoic acid. The C_9_ compound can then serve as a platform molecule for the branched C_9_–C_18_ alkanes that could be useful as jet fuel components. Alternatively, GVL can be also transformed into jet fuels via formation of C_4_ alkenes [86]. The two-step process involves an initial GVL decarboxylation over an acidic silica/alumina catalyst to generate a gas stream of butene isomers and CO_2_. In a second reactor, the butene stream is oligomerized over an acidic catalyst to yield a distribution of alkenes centered at C_12_. 

Some of the above technologies have been industrially adapted by Virent, a renewable fuel company, in a process denoted as Bioforming [110]. The process takes place in cascade and is self-sufficient in hydrogen, which is supplied by aqueous phase reforming of a fraction of the sugars. C–C coupling is carried out by aldol-condensation or oligomerization. According to Davis et al. [111], the hydrocarbon fuels produced by aqueous phase processing of lignocellulosic sugars would have a minimum selling price above $4 per gge. This price increases slightly when levulinic acid is used as a feedstock [112]. The GHG emissions of these processes depends highly on the hydrogen source. When hydrogen is obtained from natural gas sources, the GHG emissions of these processes increase significantly (49.2 g CO_2_/MJ) compared to the same process using hydrogen from the sugar reformer (15.3 g CO_2_/MJ). [111]. The aqueous phase processing of sugars and derivatives are mostly at the R&D and pilot stages of development, with Virent being the only company trying to scale up these technologies. Virent have two demonstration facilities with an overall capacity of 16,000 gallons per year of hydrocarbon fuels including jet fuels. Shell has partnered with Virent to produce gasoline, diesel, and jet fuel from biomass sugars, with hopes to have the process commercialized in 2020 [113]. The jet fuel produced by Virent is being tested against ASTM specifications [114].

## 4. Comparative of Technologies and Conclusions

The aviation industry is responsible for a significant fraction of the GHG emissions released to the atmosphere. To reduce the reliance of the aviation sector on fossil fuels and simultaneously decrease GHG emissions associated with this activity, governments are promoting directives to progressively replace conventional jet fuels with renewable fuels produced from biomass. Since oxygenate fuels such as ethanol and biodiesel do not meet the stringent requirements for jet fuels, it is imperative that the development of technologies allows the conversion of biomass and biomass feedstocks into liquid hydrocarbon fuels chemically identical to those currently used in the aviation sector. The present paper provides an overview of the main technologies available today for this purpose, with particular focus on those routes involving catalytic reactions. The main characteristics of the four routes (OTJ, GTJ, ATJ, and STJ) analyzed herein are comparatively described in Table 1.

With regard to commercial readiness, the OTJ is ahead of the other technologies, although some issues (e.g., feedstock availability, GHG emissions, lack of aromatics) need to be improved before commercialization. The GTJ route is also mature enough, with some demonstration plants currently operative. However, the high capital costs of this route (associated with the utilization of biomass as a carbon source) are important barriers hindering the commercialization of this technology. The ATJ route has already shown potential to convert any small alcohol derived from biomass into jet fuel components. While the production of the bioalcohol is technically solved, the integration of the catalytic upgrading steps (dehydration, oligomerization, and hydrogenation) with the alcohol production remains challenging. Additionally, the lack of economic and GHG data on this route avoids fair comparison with the other technologies. Finally, the aqueous-phase catalytic conversion of sugars (and platform molecules) into liquid hydrocarbon fuels has demonstrated potential at both the lab and pilot scales. The economic perspective of these routes, however, can be improved by reducing the number of processing steps required to achieve the chemical transformation.

## Figures and Tables

**Table 1:** Some features of the main routes for the conversion of biomass into jet fuels.

	OTJ	GTJ	ATJ	STJ
Feedstocks	Vegetable oils	Lignocellulose, municipal and agricultural residues,	Biomass-derived alcohols	Sugars, furanics, platform molecules
Reaction steps	Hydrotreating Fractionation	Gasification Fischer–Tropsch Fractionation	Dehydration Oligomerization Hydrogenation Fractionation	Deoxygenation C–C coupling Hydrogenation Fractionation
Catalysts	Mostly alumina-supported metal sulfides	Fe- and Co-based supported catalysts	Heterogeneous and homogeneous acid catalysts	A wide range of heterogeneous catalysts
Commercial readiness	Commercial	Demonstration	Laboratory–demonstration	Laboratory–demonstration
Minimum selling price ($/gal)	4.4–5.1 [45]	3.9–4.3 [62]	NA	Above 3.5 [111]
GHG emissions (g CO_2_/MJ)	13–141 [47]	2–10 [65]	NA	15–49 [111]
Jet fuel with aromatics	No	Yes, with FT-SKA	Yes	Yes
ASTM approved fuel	Yes, blended up to 50% *v/v* with fossil jet fuel	Yes, blended up to 50% *v/v* with fossil jet fuel	Yes, blended up to 50% *v/v* with fossil jet fuel	Test against ASTM on going
